# Toward a Digital Future in Bipolar Disorder Assessment: A Systematic Review of Disruptions in the Rest-Activity Cycle as Measured by Actigraphy

**DOI:** 10.3389/fpsyt.2022.780726

**Published:** 2022-05-23

**Authors:** Priyanka Panchal, Gabriela de Queiroz Campos, Danielle A. Goldman, Randy P. Auerbach, Kathleen R. Merikangas, Holly A. Swartz, Anjali Sankar, Hilary P. Blumberg

**Affiliations:** ^1^Department of Psychiatry, Yale School of Medicine, New Haven, CT, United States; ^2^Department of Psychiatry, University of Oxford, Oxford, United Kingdom; ^3^Interdepartmental Neuroscience Program, Yale School of Medicine, New Haven, CT, United States; ^4^Department of Psychiatry, Columbia University, New York, NY, United States; ^5^Genetic Epidemiology Research Branch, Intramural Research Program, National Institute of Mental Health, Bethesda, MD, United States; ^6^Department of Psychiatry, University of Pittsburgh Medical Center, Pittsburgh, PA, United States; ^7^Neurobiology Research Unit, Rigshospitalet, Copenhagen, Denmark; ^8^Department of Radiology and Biomedical Imaging, and the Child Study Center, Yale School of Medicine, New Haven, CT, United States

**Keywords:** bipolar disorder, actigraphy, digital technology, circadian rhythms, rest, sleep, treatment

## Abstract

**Background:**

Disruptions in rest and activity patterns are core features of bipolar disorder (BD). However, previous methods have been limited in fully characterizing the patterns. There is still a need to capture dysfunction in daily activity as well as rest patterns in order to more holistically understand the nature of 24-h rhythms in BD. Recent developments in the standardization, processing, and analyses of wearable digital actigraphy devices are advancing longitudinal investigation of rest-activity patterns in real time. The current systematic review aimed to summarize the literature on actigraphy measures of rest-activity patterns in BD to inform the future use of this technology.

**Methods:**

A comprehensive systematic review using PRISMA guidelines was conducted through PubMed, MEDLINE, PsycINFO, and EMBASE databases, for papers published up to February 2021. Relevant articles utilizing actigraphy measures were extracted and summarized. These papers contributed to three research areas addressed, pertaining to the nature of rest-activity patterns in BD, and the effects of therapeutic interventions on these patterns.

**Results:**

Seventy articles were included. BD was associated with longer sleep onset latency and duration, particularly during depressive episodes and with predictive value for worsening of future manic symptoms. Lower overall daily activity was also associated with BD, especially during depressive episodes, while more variable activity patterns within a day were seen in mania. A small number of studies linked these disruptions with differential patterns of brain functioning and cognitive impairments, as well as more adverse outcomes including increased suicide risk. The stabilizing effect of therapeutic options, including pharmacotherapies and chronotherapies, on activity patterns was supported.

**Conclusion:**

The use of actigraphy provides valuable information about rest-activity patterns in BD. Although results suggest that variability in rhythms over time may be a specific feature of BD, definitive conclusions are limited by the small number of studies assessing longitudinal changes over days. Thus, there is an urgent need to extend this work to examine patterns of rhythmicity and regularity in BD. Actigraphy research holds great promise to identify a much-needed specific phenotypic marker for BD that will aid in the development of improved detection, treatment, and prevention options.

## Introduction

Bipolar disorder (BD) is a mood disorder characterized by episodes of depression and mania, interspersed with periods of relative stability termed euthymia (1). Based on the American Psychiatric Association’s (APA) Diagnostic and Statistical Manual of Mental Disorders (DSM-5; 2), BDI is diagnosed if an individual has experienced a manic episode, while BDII is diagnosed when elevated mood symptoms have only reached the level of hypomania. Individuals with BD often spend more time in depression. Course features such as the frequency of episodes, and clinical features such as the presence of psychotic symptoms and comorbidities, vary among individuals with the disorder. Given the clinical heterogeneity of BD, markers for early identification, differentiation from related mood disorders such as major depressive disorder (MDD), and discovery of novel treatment targets are vital. Notably, criteria for the hallmark manic and hypomanic episodes include increases in activity levels and decreases in sleep though individuals may feel rested. These features suggest the potential importance of the study of activity and rest patterns in elucidating the pathophysiology of BD and in improving detection and interventions.

### Rest-Activity Patterns in Bipolar Disorder

Prior to the advent of actigraphy research in BD, in addition to sustained changes in emotional states, disturbed sleep and activity patterns were observed clinically to also be core features of acute episodes of BD. These patterns present differently depending on mood states and can be predictive of both onset and severity of illness (3). Daily rhythms are central to the presentation of both depression and mania, such that changes in both sleep and activity levels play a prominent role in the criteria for acute episodes of BD as outlined in the DSM-5 (2). Sleep problems present in depressive episodes in the form of insomnia or hypersomnia in the majority of cases (4), whereas hypo/manic episodes are often characterized by marked reductions in sleep accompanied by subjective reports of not feeling tired (5). Further, individuals with BD, even when euthymic, have been observed to show delayed onset of the sleep-wake phase and lower levels and later timing of melatonin secretion, an important hormone in circadian rhythm regulation (6–8). Successful chronotherapeutic interventions that modify rest-activity patterns in BD have been reported (9–11), suggesting that they constitute promising intervention targets.

Disruptions in rest-activity patterns have been previously linked to the onset of BD episodes, such that hypomania and mania has been reported following a night of sleep deprivation (12). Pinho et al. (13) found a significant relationship between disruptions in patterns of daily activity and sleep and the severity of depressive symptoms, such that the degree of disruption in patterns was associated with psychosocial dysfunction. The persistence of disruptions during euthymic periods, albeit at a lower magnitude, also suggests that this feature of BD can persist outside of syndromal mood episodes and might be a trait feature of the disorder. During euthymia, individuals with BD have shown reduced daytime activity (14, 15) and greater intra-daily variability of rest-activity patterns (15, 16) compared to healthy controls (HCs). Difficulty in falling and staying asleep (17–19) and early morning awakening persist during euthymic periods (20). Additional studies suggest that disturbed rhythms during euthymia may relate to vulnerability for future episodes and adverse outcomes as they have been associated with a greater severity of subsequent depressive and manic episodes over a 12-month period (21), a history of psychosis, and suicide attempts (22).

### The Importance of Capturing Both Rest and Daily Activity

Biological processes that underlie circadian rhythms are implicated in the sleep-wake cycle. Quantifiable proxy markers for this cycle are rest and activity patterns, both of which, notably, can influence each other. Holistic examinations of disturbance across the 24-h cycle, not just during the rest period, (23–26), are therefore warranted. This comprehensive approach to rhythmicity is further reflected in the DSM-5 diagnostic criteria for episodes of BD which include increased energy or activity alongside decreased need for sleep in manic episodes, and psychomotor retardation and loss of energy alongside insomnia or hypersomnia in depressive episodes (2).

### Traditional Methods for the Assessment of Rest-Activity Patterns

Sleep-specific measures and measures of the peripheral neurohormone, melatonin, have largely been employed in past studies of the sleep-wake cycle in persons with mood disorders. Polysomnography (PSG) and sleep diaries have traditionally provided objective and subjective information about sleep patterns, respectively. These methods support clinical reports of disrupted sleep patterns in BD across mood states and in comparison to both HCs and those with MDD [characterized by periods of depression in the absence of hypo/mania] (27, 28). However, these methods provide only limited data about activity during wakefulness, and while sleep diaries may also include questions about activity, they are often limited in scope and rely on subjective responses. Nevertheless, correcting melatonin abnormalities has been suggested to be the mechanism underlying the therapeutic effect of mood-stabilizing pharmacotherapies. For example, lithium and sodium valproate have been shown to reduce melatonin suppression, thus increasing overall melatonin levels (6, 29), and potentially targeting mania vulnerability (30). Outside of pharmacological interventions, chronotherapies such as bright light therapy, which is known to influence melatonin secretion, have been shown to be clinically effective in depression by advancing the phase of the sleep-wake rhythm (10, 31).

### The Future of Rest-Activity Pattern Assessment in Bipolar Disorder: Actigraphy

In order to better understand BD, digital technologies have been leveraged to noninvasively collect biometric parameters when individuals are in their everyday environments (32, 33). Temporal profiles of rest and activity, measured with digital actigraphy methods, hold promise for elucidating a novel phenotypic marker for BD (34, 35). Identification of a salient time-varying phenotypic marker for BD may also reveal pathophysiological mechanisms of BD, which ultimately could serve as the basis for novel detection, treatment, and prevention strategies. Prior methods used to characterize the time-varying patterns of BD, such as retrospective self-reports, are limited in their ability to provide accurate data at high levels of granularity (36). The high temporal resolution of actigraphy offers an alternative approach to capturing the time-varying characteristics of BD, and over time, may lead to advancements in early identification and treatment.

Thus, actigraphy assessment of the rest-activity cycle addresses the need for longitudinal and temporally sensitive objective assessments in individuals’ natural environment. Wristwatch-like wearable actigraphy devices assess gross motor activity using measures of acceleration speed *via* accelerometers, providing low cost and non-invasive rest-activity assessments (37). It is important to note that actigraphy does not measure sleep directly, but rather uses movement as a proxy for estimating sleep, although terminology used in previous publications often include the use of the word “sleep.” Parameters estimated from actigraphy data include the duration of rest or sleep periods, activity levels during rest or sleep periods, time in bed (TIB), total sleep time (TST), sleep onset latency, and daytime naps (38). Table 1 describes common actigraphy parameters in more detail. Measurements are time-stamped and made continuously, often at high measurement frequencies, for example, every 1-min. This data collection format facilitates data visualization using readily available software packages. Actigraphy has a high rate of concordance with PSG and sleep diaries in healthy populations (39–41) and in individuals with BD. For instance, similar patterns of sleep and rest quality and duration have been reported across the three measures, showing high levels of inter-correlation (42) in BD (42–45), and the ability of actigraphy to sensitively distinguish between BD depressive, manic, and euthymic episodes (46).

**TABLE 1:** Overview of commonly used actigraphy parameters.

	Variable name	Description
Rest	TST	Total sleep time. The time between sleep onset and offset/final wake time, minus any periods of wakefulness in between (WASO).
	TIB	Time in bed. The duration spent in bed, calculated as the time between going to bed and arising, usually aided by pressing an “event marker” button on the actigraph, if available.
	WASO	Wake after sleep onset. The total number of nocturnal waking minutes.
	Sleep efficiency (SE)	Percentage of time asleep between sleep onset and offset/final wake time.
	Sleep onset latency (SOL)	The number of minutes between bedtime and sleep onset.
	Fragmentation index (FI)	The amount of movement or restlessness in a rest period.
	L_5_ onset	Onset of the lowest 5 h of activity in a 24-h period. A proxy marker for sleep/rest onset.
	L_5_ activity	Activity levels over the lowest 5 h of activity in a 24-h period, after L_5_ onset. A proxy marker for sleep/rest activity.
Daytime activity	M_10_ onset	Onset of the most active 10 h in a 24-h period. A proxy marker for day/activity onset.
	M_10_ activity	Activity levels over the greatest 10 h of activity in a 24-h period, after M_10_ onset. A proxy marker for day/diurnal activity.
Rest-activity rhythms	Relative amplitude (RA)	Differentiation score of activity during the ten most active hours in a 24-h period (M_10_ activity) compared to activity during the five least active hours in a 24-h period (L_5_ activity). Therefore, differentiation in activity during active and rest states. Scored between 0 and 1, with a lower RA representing lower differentiation.
	Intradaily variability (IV)	A variability marker of the difference in patterns within a day. Greater values represent greater rhythm fragmentation. Greater fragmentation indicates more transitions between rest and active states.
	Interdaily stability (IS)	A stability marker of the difference in patterns across days. Greater values represent greater stability of rhythm. Greater stability indicates more consistency of rest-activity patterns between days.

Notably, while actigraphy is effective in estimating rest-activity patterns, it does not provide deeper measures of sleep such as brain activity (EEG), heart rhythm (ECG), or eye movements (EOG) that are provided by PSG (47). Therefore, actigraphy cannot characterize sleep physiology and sleep architecture. Actigraphy data also requires careful interpretation as, for example, time resting in bed could be mistaken for sleep time. For that reason, referring to the period encompassing sleep as “rest,” or the “rest period,” is considered most appropriate for actigraphy measures. Furthermore, it is important to distinguish circadian rhythmicity from 24-h activity patterns. Certain actigraphic measures, such as interdaily stability (IS), relative amplitude (RA), and intradaily variability (IV), are often used erroneously to characterize endogenous circadian function (48). Similarly, L_5_ onset and M_10_ onset are also often used as markers for circadian phase timing, and changes to these variables are often erroneously concluded to be phase advances or delays. While these measures provide information about 24-h activity patterns, rhythm stability, and fragmentation (see Table 1), they must not be interpreted as markers of endogenous function. Therefore, actigraphic constructs are discussed here in the context of rest-activity patterns. But it should be noted that rest-activity patterns may serve only as a proxy measure for the sleep-wake cycle, which in turn, may serve as a proxy measure of overall circadian function. It is important to note that the sleep-wake cycle is one of a number of physiological, behavioral, and cognitive functions under partial control of the circadian system, and thus, a disruption in the rest-activity or sleep-wake cycle does not necessarily equate to underlying circadian rhythm dysfunction.

### The Current Systematic Review

The current review aims to summarize actigraphy assessments in BD published to date, encompassing both rest and activity profiles. As a key goal is to review evidence supportive of the potential clinical significance, special focus is given to findings that help to elucidate (a) the ability of these patterns to distinguish BD from comparison groups, including HC and other clinical groups; (b) the relationship these patterns have with distinct mood states within BD and with other clinical, cognitive, and brain features of the disorder; and (c) the propensity for actigraphy to decipher the therapeutic effects of treatment interventions on mood measures in BD. We therefore address three research questions: (a) what are the rest-activity patterns associated with adult BD and how do they differ from those in HCs and other clinical comparison groups? (b) what relationship do these patterns have with other characteristics of BD, including mood, cognition, and neurobiology? (c) what effect do interventions, encompassing both pharmacological and non-pharmacological therapies, have on these patterns in BD?

Actigraphy measures have previously been employed in empirical studies of BD for at least 15 years, with a number of systematic reviews measuring their ability to capture rest-activity disturbances in those with BD [e.g., (17, 49)]. However, the clinical utility and significance of this method has not yet been assessed, and thus the translation of this method to clinical practice has not yet been widely achieved. This is increasingly important given the recent shift toward digital and longitudinal methods that are adept at assessing temporally sensitive mood disorders. Therefore, the current review aims to show how actigraphy technology can pave the way for better capturing the temporally dynamic nature of BD, and ultimately aid in the discovery of mechanisms underlying BD and more nuanced targets for detection, treatment, and prevention.

## Methods

### Data Sources

Studies were independently identified by authors PP and GdQC through manual searches of the electronic databases PubMed, MEDLINE, PsycINFO, and EMBASE. While a broad systematic method was used, following PRISMA guidelines (50), emphasis was placed on a thorough literature search of actigraphy studies in BD. To this end, the following terms were used in our primary search (further detailed in Supplementary Table 1): “actigraphy,”, “actigraph,” “actimetry,” “accelerometry,” “smart watch,” or “health watch” (separated by OR) in combination (using AND) with the terms “BD” and “bipolar” (separated by OR). Only research-grade actigraphy devices were searched for (excluding commercially available devices such as FitBit or Apple Watch) in order to ensure data reliability and validity, based on clinical specificity issues (51). Eligible papers included studies of BD samples. No lower publication date limit was applied, and the search was continued until 19th February 2021. All selected articles were published in peer-reviewed journals in English.

### Data Extraction

Information extracted from identified manuscripts were sorted into four categories: study details (author names, year of publication, and title); demographic and clinical details of subject samples [number of participants, age (mean and standard deviation), gender, diagnosis category, mood status (i.e., depressed, manic, and euthymic), and medication status]; actigraphy methodology (device name, body location, recording time or duration, and sampling/recording rate used); and main study outcomes (daily activity, rest/sleep, and 24-h patterns as outlined in Table 1). Data was independently and manually extracted by PP and GdQC by going through each article and Supplementary Material, if available. Any discrepancies were discussed, checked, and cleared by the authors.

In order to capture all relevant cross-sectional and longitudinal studies using actigraphy, BD subtypes BDI, BDII, and BD NOS or OS [(not) otherwise specified, i.e., with symptoms of BD that do not meet full diagnostic criteria] under both DSM (2) and International Classification of Diseases (52) diagnostic manuals were included. All pharmacological and non-pharmacological interventions employed in actigraphy studies of BD were included to address the third research question. Overall comparators included HCs, those with related psychiatric disorders [MDD, anxiety disorders, schizophrenia, borderline personality disorder (BPD)], insomnia, or general mental health service users. Actigraphic outcomes relating to patterns over 24-h, daytime activity, and markers of rest and nighttime period were used as outcomes.

Articles were excluded from the final list during the manual data extraction process if they aimed to validate methods (i.e., using actigraphy to validate against PSG), focused on the study of children and adolescents (defined as studies that included individuals under 18 years of age) or were solely conducted in at-risk groups, case studies, descriptions of study protocols, or genetic studies.

### Research Questions

In order to address the avenues of investigation of the rest-activity patterns measured through actigraphy, the review was organized by three research questions. First, what are the rest-activity patterns associated with adult BD and how do they differ from those in HCs and other clinical comparison groups? Second, what relationship do these patterns have with other characteristics of BD, including mood, cognition, and neurobiology? Third, what effect do interventions, encompassing both pharmacological and non-pharmacological therapies, have on these patterns in BD?

## Results

### Study Selection

**FIGURE 1:**
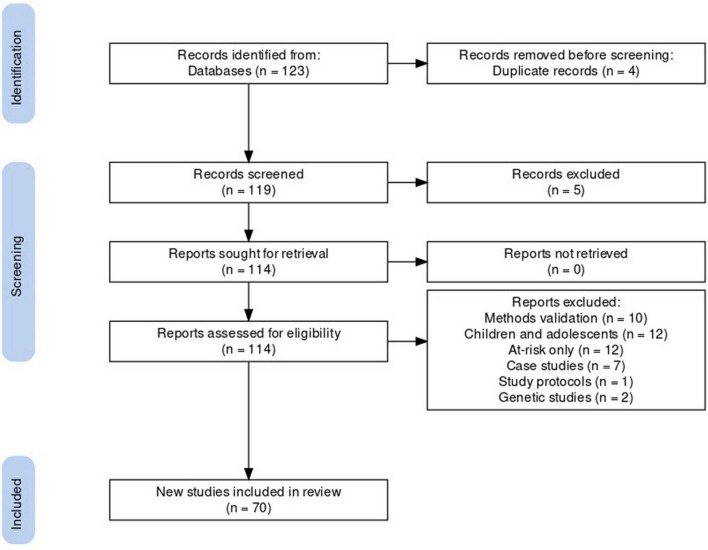
Study selection flow chart.

The database search identified 114 unique articles, of which 70 met systematic review criteria (Figure 1). Articles that were excluded from the final list fell into the following categories: methods validation (*n* = 10), the study of children and adolescents (*n* = 12) or at-risk groups (*n* = 12), case studies (*n* = 7), study protocols (*n* = 1), or genetic studies (*n* = 2). To address the three research questions: 41 of the 70 included articles compared actigraphy in BD with that of HCs or other clinical groups; 19 investigated the relationship between rest-activity patterns and different features of BD; and 10 articles assessed the effect of interventions on actigraphy measures in BD. Study characteristics are presented in Supplementary Table 2. All rest and activity pattern findings reported in section “Results” are derived from the 70 actigraphy studies.

### Consistency in Methods

As demonstrated in Supplementary Table 2, actigraphic recording periods varied greatly such that studies collected data from anywhere between 24 h [e.g., (53–55)] and 90 days (56). While 44% (*n* = 32) of studies accounted for the 24-h nature of rest-activity patterns, a further 39% (*n* = 28) focused on recordings during the rest or nighttime period only, and 17% (*n* = 12) focused on those of daytime activity only.

Given the small number of studies and methodological heterogeneity in the studies identified (as evidenced by varying sample sizes, length of actigraphic recording, devices used, and variables extracted), we did not calculate effect measures for each outcome. The studies were not directly comparable enough to mathematically assess meaningful differences between them. Our results below are based on the findings of each of the studies included in this systematic review.

### Question 1: What Are the Rest-Activity Patterns Associated With Adult Bipolar Disorder?

Of 41 articles addressing this question, 49% (*n* = 20) directly compared rest-activity patterns in persons with BD with those of HCs and 37% (*n* = 15) included other psychiatric clinical comparison groups, 5% (*n* = 2) included persons with a diagnosis of insomnia, 2% (*n* = 1) included persons with childhood attention deficit hyperactivity disorder (ADHD), and 7% (*n* = 3) included at-risk groups [i.e., non-affected siblings or first or second degree relatives, and those with a previous episode of major depression with sub-threshold mania symptoms (57)] in addition to BD and HC groups.

#### Rest

In BD, compared to HCs, longer sleep latency and longer sleep duration were most often reported (58–63) and were associated with symptoms of depression (64). Although reported less often, later sleep midpoint was also noted (65). Gershon et al. (60) found that increased sleep latency, as well as wider disruptions in rest continuity including longer wakefulness after sleep onset (WASO) and lower sleep efficiency (SE), were associated with increased negative affect in euthymic BD. They did not detect associations between these rest measures and levels of positive affect in their sample, suggesting the connection between these rest patterns with depression but not mania. Notably, there was a difference in subjective and objective measurements of these rest parameters such that Krishnamurthy et al. (66) found that individuals with BD, compared to HCs, had higher absolute discrepancy between objective and subjective sleep latency, i.e., the BD group self-reported even longer sleep latency than suggested by actigraphy measures, and between subjective and objective TST, i.e., those with BD self-reported significantly lower sleep duration compared to their actigraphy measurement. They, and Gonzalez et al. (67), found that the severity of depressive symptoms was also associated with greater discrepancy between subjective and objective measures of rest, implicating mood state not only in the timing of rest in BD but also in its perception.

To determine whether actigraphy patterns are specific to the depression of BD, studies have also included individuals with MDD, although they have been sparse and inconclusive to date. Robillard et al. performed a series of studies that identified both sleep pattern-related features that were common across depression in BD and MDD, and others that differed suggesting specificity to BD-depression. They observed lower TST and more irregularity of circadian patterns, as measured by a circadian rhythmicity index, to be predictive of worsening verbal memory across groups, suggesting these patterns may be more associated with this cognitive feature in depression than diagnosis (68). However, they observed sleep phase to be more delayed and sleep offset (time of awakening after sleep) to be later in BD-depression than MDD-depression, and that lower SE was predictive of later worsening in manic symptoms (7, 69). In a study that compared BDI, BDII, and MDD, Shou et al. (15) found differences in the average and variability of these subgroups at different times of the day compared to controls. Whereas people with BDII had greater variability during the rest period, those with BDI had lower levels of and greater variability in activity later in the day. These findings also showed specificity for BD because those with MDD did not differ from controls in either average or variability in motor activity at any of the specific time periods or across the whole day (15). These studies suggest that actigraphy may identify specific aspects of current and impending mood symptoms of BD.

A small set of studies compared BD and HC groups to those at risk of developing BD, due to family history or subsyndromal symptom presentation. These studies reinforce the notion of a link between rest disturbances and BD vulnerability. Verkooijen et al. (70) found that later sleep offset (i.e., later time of awakening after sleep) and longer sleep duration across individuals with BD and their non-affected siblings were associated with increased depressive symptoms, suggesting a link between sleep pattern rhythmicity and vulnerability to depression. Ritter et al. (57) observed prolonged sleep latency and difficulties awakening, in both euthymic individuals with BD and those at risk, suggesting that these sleep rhythmicity disturbances may constitute a BD trait. These patterns were significantly different from those in HCs, suggesting that disturbance in rest periods is a characteristic of BD, and could be both an early risk marker for its development and a potential target prior to the initial manic episode. Longitudinal studies are needed to determine whether the rest findings are associated with the development of BD in youths at risk.

Research comparing BD with other clinical groups suggests that diagnostic group status can be predicted with a high degree of accuracy from rest data estimated from actigraphy alone (71). Although studies have suggested that rest disturbances in BD and MDD are quite similar, indicating that sleep fragmentation is an indicator of depression in both groups (72), other research has been able to use actigraphy to distinguish BD from other clinical groups. For instance, delayed onset of rest patterns, as measured by L_5_ onset (see Table 1), was associated with BPD rather than BD, despite equal levels of depression (73). Of note, a history of ADHD symptoms (in adults with a childhood history of ADHD) was not found to be associated with rest period disturbances in BD (74).

#### Daytime Activity and Rhythmicity

Despite a limited number of articles on daytime activity levels, findings suggest that abnormal daytime activity patterns are characteristic of BD. In comparison to HCs, individuals with BD who are euthymic and those who have hypo/manic or depressive symptoms have shown lower average daily activity, more periods of daytime napping, and decreased circadian RA, i.e., less differentiation between daytime and nighttime activity levels (16, 54, 75). Studies suggest that the general lowering of daytime activity in BD could be used as a target for therapy (76).

Individuals with BD, compared to HCs, showed higher variability in activity over 24 h (15, 54). In individuals experiencing mania, this increased variability occurred primarily in the morning period, as they displayed reduced autocorrelation during this time (lower correlation of activity counts between subsequent minutes of data collection), differentiating it from depression (77, 78). Of interest, different patterns of variability of activity have been found at different times of the day for BDI and BDII subgroups compared to controls or people with MDD (15). Additionally, compared to HCs, BD mania was associated with a phase advance of around 7 h, while depression was associated with a phase delay of 4 h (79), supporting mood state-related differences in the time of day of activity disturbances. In sum, these studies highlight the importance of timing in the variability of activity, suggesting that timing of activity regulation could be a target for therapy.

Actigraphy differences are also seen when comparing activity patterns in BD to other clinical groups. For instance, similar to findings in comparisons to HC groups, individuals with BD have shown significantly less daytime activity than other mental health service users (80). BD has also been more strongly associated with lower daytime motor activity compared to MDD (81). Individuals with schizophrenia, compared to individuals with BD, had a more complex profile of activity showing more variability at a smaller time scale (54, 77, 78). However, a study of the onset of daytime activity (as measured by M_10_ onset, see Table 1) did not distinguish BD from BPD, as both groups had significantly later onset compared to HCs, but did not differ from each other (73, 82). These data suggest that daytime activity patterns may differentiate BD from other major mood and psychotic disorders, although it may be more difficult to distinguish from BPD.

#### The Contribution of Machine Learning Methods

Building on these findings of differential activity patterns during rest and daytime periods in BD, researchers have also begun to use novel machine learning methods to classify individuals with BD and HCs using actigraphy data, such as 24-h patterns. For instance, using the random forest classifier method, Schneider et al. (56) was able to correctly classify euthymic BD and HCs with a sensitivity of 85% and a specificity of 91%. Krane-Gartiser et al. (83) used a combined rest and activity model to correctly classify 75% of individuals with BD. These tools may also be used to investigate the extent to which the actigraphy features exhibit significant interaction and correlation both within and between domains. For example, Di et al. (84) have applied a dimension reduction technique, Joint and Individual Variation Explained (JIVE), that efficiently deals with multivariate data representing multiple domains that may help to hone in on the core rhythmic patterns underlying BD. Future studies emphasizing longitudinal actigraphy monitoring may be able to better train machine learning algorithms to perform these classifications. This supports the value in using such methods and highlights the potential for such classification tools to be used alongside traditional diagnostic measures.

### Question 2: What Relationship Do Rest-Activity Patterns Have With Other Characteristics of Bipolar Disorder?

Nineteen of the 70 included articles addressed this research question. Of these articles, 43% (*n* = 8) addressed the relationships between rest-activity patterns and specific mood states, 21% (*n* = 4) investigated the neuroimaging correlates of these patterns, as measured by magnetic resonance imaging, 10% (*n* = 2) studied the relationship with suicidal thoughts and behaviors, 10% (*n* = 2) assessed the association with cognition, 6% (*n* = 1) investigated the relationship with metabolic symptoms, and an additional 10% (*n* = 2) assessed the relationship between these patterns and coffee, tobacco, and alcohol consumption (85), and characteristics of social support and strain (86).

#### Rest

Investigations focused exclusively within BD, aiming to understand the relationship between rest patterns and clinical features of BD such as its different mood states, remain limited. Krane-Gartiser et al. (87) found that mood variability in BD during 1 week, measured as significant change on a mood scale adapted from the Systematic Treatment Enhancement Program for Bipolar Disorder study (88), was significantly associated with rest period disturbance. Those in the unstable mood group presented with delayed sleep phase, as well as later and more variable bedtimes and awakening times. Further, Kaplan et al. (89) identified two distinct subtypes of hypersomnia in euthymia using actigraphy: one characterized by “long sleep” or a long TIB rather than a long sleep duration, and the other by “excessive sleepiness.” The latter was able to predict later relapse to mania or hypomania. These findings suggest that changes in rest patterns are not only associated with depressive symptoms but may also reflect a wider disruption in underlying neurobiological regulatory mechanisms that may contribute to vulnerability to subsequent episodes.

Notably, disruptions in the rest period have shown associations with adverse outcomes, including preventable risk for early mortality, highlighting the importance of studying rest period disturbances in BD to improve prognosis. Difficulty falling and staying asleep, as well as fragmented sleep, have been associated with a greater history of suicide attempts in euthymic individuals with BD (90) and with suicidal ideation across euthymic, depressed, and hypomanic individuals with BD (91). Associations between fragmented sleep, as well as poor SE, with increased cardiovascular metabolic risk factors have also been found in euthymic BD (92). Environmental and psychosocial factors may be important targets for prevention in euthymia as circadian disruptions are linked to coffee, alcohol, and tobacco consumption (85), and Eidelman et al. (86) found a relationship between instability in rest periods and lack of social support in BD.

Study of associations between actigraphy with cognitive and neuroimaging measures can help to uncover the neurobehavioral mechanisms that contribute to mood and rest patterns. There is growing evidence that disturbances in the rest period in BD, involving both the onset and duration of sleep, are associated with impaired white matter microstructure, as measured by lower fractional anisotropy, particularly in the genu and body of the corpus collosum and corona radiata (93). Additionally, disturbances in SE and abnormal activity rhythms in euthymic BD were found to be associated with increased functional connectivity in the dorsolateral prefrontal cortex, implicated in working memory processes (94). Though not based on actigraphy measures, a study of cognition in euthymic BD reported significant associations among subjective sleep disturbances, cognitive impairment, and poor work-related outcomes (95). The association between rest and cognition also seems to be linked to specific working memory and attention domains, supported by the neuroimaging associations reported by McKenna et al. (94). Further, Bradley et al. (96) also found that euthymic individuals with BD with abnormal rest patterns performed poorly on tests of sustained attention and working memory compared to those with normal rest patterns and HCs.

#### Daytime Activity and Rhythmicity

Studies that assess daytime activity patterns within BD report different patterns of activity as indicators of mood state. In Gershon et al. (97)’s study of inter-episode BD, depressive states were found to be distinguished from other mood states by even lower levels of activity, and a later time of activity onset (M_10_ onset). Other studies have reported associations between more irregular activity patterns and a greater severity of manic symptoms (67, 98), and higher baseline activity patterns in BD have been associated with relapse into mania or hypomania (99). Building on this by using predictive modeling, Scott et al. (100) found that daytime activity parameters, including absolute activity, variability markers, autocorrelation, and patterns of regularity, were able to correctly classify BD cases into distinct mood episodes, with particularly high rates for both mania and mixed states. These data suggest that changes in motor activity may be sensitive markers of mood state that could be useful in identifying relapse and measuring treatment response, and that further study of activity patterns in BD may help in the elucidation of mechanisms underlying mood states.

Daytime activity patterns in BD have been associated with adverse clinical outcomes. For example, an earlier onset of daytime activity, as measured by M_10_ onset, suggesting a shortened or disrupted period of rest, was associated with a history of increased suicide attempts in euthymic individuals with BD (90). Further, fragmented profiles of activity as measured by RA and rest duration, were shown to be associated with higher systolic blood pressure (92) and increased alcohol consumption (85). Thus, disrupted daytime activity levels could be indicators of risk for worsening outcomes not only of BD but also the well-established links with cardiovascular disease (101) and substance use disorders (102). These associations with comorbid conditions warrant further investigation through longitudinal studies.

When exploring how daytime activity relates to brain structure and function, studies have found associations with white matter microstructure. An association between lower activity levels with fractional anisotropy in the left bilateral corticospinal tract, a region of the motor pathway, was suggested as a compensatory mechanism for illness-related psychomotor retardation during depressive mood states (103). Verkooijen et al. (93) reported an association between stability in activity patterns with increased fractional anisotropy, particularly in the genu and body of the corpus callosum, and the right anterior corona radiata, which provide connections in brain systems that subserve emotion regulation and behavioral control. Although these findings were not specific to the BD group and were also reported in HCs, they suggest potential links between stability in activity patterns measured by actigraphy and the structural integrity of white matter in brain connections related to motor activity, and emotional and other behavioral regulation. When examining resting state cerebral perfusion, a relationship between cerebral blood flow and daytime activity levels in both euthymic BD and MDD has been reported (104). In both MDD and BD, reduced activity levels were associated with alterations in the middle frontal gyrus and insula, regions implicated in cognitive and emotional functions. In the BD group only, this relationship was further noted in the left precentral gyrus, which subserves motor function (104). Together, these results begin to highlight relationships between patterns of cognitive and emotional processing and motor activity with rest-activity patterns in BD. However, given the small number of studies that have currently investigated these patterns, further investigation combining actigraphy and neuroimaging methods is needed.

### Question 3: What Effect Do Interventions Have on Rest-Activity Patterns in Bipolar Disorder?

Ten of the 70 included articles addressed this research question. Of these articles, 60% (*n* = 6) investigated differences in rest-activity patterns after pharmacological treatment including mood-stabilizing medications (lithium and quetiapine), and 40% (*n* = 4) investigated differences in these patterns after non-pharmacological treatments including light therapy, cognitive behavioral therapy (CBT), and blue-blocking (BB) glasses. Risk of bias assessment (105, 106) was completed on these interventional studies and can be found in Supplementary Figure 1.

#### Rest

When investigating the response to lithium, a long-standing mood-stabilizing pharmacological treatment for BD, individuals who responded well as assessed by the Retrospective Assessment of Response to Lithium Scale (the Alda scale) were also found to have more regular rest-activity patterns following treatment, as measured by IV and RA (107). Further, when using principal components analysis to classify lithium responders based on their actigraphy parameters, the same investigative group (108) found that circadian rhythmicity markers involving regularity and stability in patterns (RA, IV, IS, as well as M_10_ activity) were able to correctly classify 64% of BD cases as good responders as determined by the Alda scale.

In comparisons of lithium to that of the antipsychotic quetiapine, the latter was associated with improvements in objective sleep parameters, including sleep quality, SE, and WASO, over and above lithium (109). These findings have yet to be replicated. Other studies assessing the role of quetiapine have found a relationship between rest-activity patterns and later improvements in depression. For instance, Todder et al. (110) found that a rapid and consistent improvement in objective rest parameters (as measured by sleep latency) was observed after 1 week of quetiapine treatment in BD-depression, but that these improvements were not related to changes in depression symptoms at the same time. The researchers found that these objective improvements at week 1 instead predicted longitudinal improvements in depression scores after 4 weeks (111). These results provide objective data supporting longstanding clinical observations that some of the earliest changes during recovery from depressive episodes are changes in activity (112) and that emotional state changes may require additional weeks. This suggests that actigraphy measures of rest patterns may be important in detecting early indicators of improvements in depressive symptoms and antidepressant effects.

A small number of studies have investigated the effect of non-pharmacological therapies targeting rest-activity patterns, including bright light therapy and sleep deprivation therapy. The aim of these therapies is to stabilize or reset the circadian rhythm, either by changing its phase or increasing rhythm amplitude, with the goal of improving symptoms. In Benedetti et al. (113)’s study of individuals with BD depression treated with sleep deprivation therapy alongside morning light therapy for 1 week, two-thirds of subjects responded with a 50% reduction in depressive symptoms and a circadian phase advance. Esaki et al. (114) showed that the timing of light therapy might be relevant to its reported effects on improving rest patterns, as they observed that light exposure at night led to decreased levels of sleep quality – including lower SE, longer sleep onset latency, delayed sleep midpoint, and greater WASO – after 1 week in individuals with BD. The use of blue-light-blocking (BB) glasses (115, 116) has been suggested as a potential treatment to oppose the effect of late night light on circadian patterns in BD. Henriksen et al. (117) found significant improvements in SE, sleep fragmentation, and fewer nights of interrupted sleep, in their BD manic sample treated with BB glasses. Lastly, one study assessing cognitive behavioral therapy for insomnia (CBT-I) with an added component to target sleep inertia (the transitional state between sleep and wake) was found to significantly improve subsequent inertia in euthymic BD (118).

#### Daytime Activity and Rhythmicity

In a study of lithium and quetiapine in individuals with BD depression, acrophase – or the timing of the peak circadian phase – was found to be delayed by both medications, but the delay was particularly significant for the quetiapine-treated group (119), consistent with previous evidence of a phase-delaying effect of lithium and quetiapine (120, 121). Quetiapine was found to have a more robust effect in shifting this circadian phase over a period of 8 weeks, and, while it seems counter-intuitive that a phase-delaying effect would improve depressive symptoms, this was associated with a reduction in depressive scores (119). These results suggest a related mechanism of regulating depressive mood and stabilizing rest-activity patterns after therapy.

When looking at actigraphy-measured markers of daytime activity during non-pharmacological therapies, the results show a therapeutic benefit but with some differences from pharmacotherapies in the patterns that they alter. For instance, in the two-thirds of individuals with BD depression who responded to sleep deprivation and light therapy over 1 week, Benedetti et al. (113) found that they showed an increase in daytime activity and also a phase advance in the rest-activity rhythm of 57 min. This phase advance after non-pharmacological treatment was also shown in a study of CBT-I with an added component to target sleep inertia, whereby individuals with euthymic BD showed an increase in morning activity levels and improved sleep inertia (118). The therapeutic effects of pharmacotherapies or non-pharmacological therapies associated with phase delays or phase advances suggests the complexity of understanding the effects of differential phase shifting.

## Discussion

### Summary

Results from this systematic review provide evidence that BD is associated with disruptions in rest-activity patterns including longer sleep duration, longer sleep onset latency (58–63), and lower average daily activity (16, 75) that may be especially associated with depression. BD mania is instead linked to more complex and variable patterns over a shorter temporal scale that can also be predictive of future relapse (54). Different mood episode types are also associated with different profiles of phase-shifting, such that a phase delay is more commonly associated in BD-depression and a phase advance in BD-mania (79). Disruptions in rest-activity patterns are shown to persist during euthymia [e.g., (14, 16, 89)] and there is preliminary evidence that distinct patterns may differentiate BD from other major mood and psychotic disorders, and within subtypes of BD (7, 69, 81). Both pharmacological and non-pharmacological therapies have demonstrated rest-activity pattern stabilization associated with improvements in mood symptoms [e.g., (107, 113)]. However, the mechanisms by which these occur are yet to be fully uncovered. This may be further facilitated by technological and digital advancements provided by actigraphy, allowing the collection of fine-grained, longitudinal, and temporally sensitive parameters while individuals are in their typical environments, and emerging machine learning and other computational strategies to extract maximal information from time series data. The extant literature supports the promise of actigraphy to pave the way for the development of improved early detection, treatment, and prevention methods.

It is important, however, for current limitations to be addressed, including variability in methodology (e.g., the actigraphic variables chosen for analyses, actigraphic recording periods, and small sample sizes) and study sample characteristics (e.g., medication use and age composition) which can impede further meta-analytic work (122). Additionally, future studies should account for variability in daily patterns due to societal demands (e.g., shift work), in order to consider how such circadian misalignment may lead to artificially fluctuating levels of actigraphic fragmentation (123) that are not representative of rest-activity patterns or endogenous circadian rhythms (124). While meta-analytic work could provide a statistical summary of results, the disparities in study characteristics did not allow for this type of analysis. It is also important to note the inherent limitations in systematic review methodology. This systematic review might exclude relevant literature as, inherent in a systematic review approach, it is bounded by search terms. Of note is the recent expansion in health-related digital technologies available for use on both the research and personal-use market. While we chose not to include commercially available digital technologies in our current review due to their limited specificity and accuracy in reporting rest-activity patterns compared to research-grade devices (51), future research could expand on this as the devices become more ubiquitous in the general population and advancements are made in optimizing their research and clinically oriented utility.

### Treatment Implications

Future actigraphy research in BD could not only benefit the work of researchers aiming to further elucidate the neurobiological basis of BD, but also clinicians and patients, who may in the future be able to use real-time and longitudinal monitoring to identify personal rest-activity patterns and warning signs (35, 125). Traditional modes of in-person clinical appointments at the temporal scale of weeks or months can benefit from the use of daily and on-going monitoring through digital technologies and passive activity sensors for their ability to provide longitudinal and prospective data, including interim data that may indicate need for more acute care (34). The use of digital technologies, such as smartphone-based ecological momentary assessment in combination with actigraphy, is gaining traction for its ability to provide complementary information on day-to-day fluctuations in mood and other symptoms, social and other daily functioning, as well as changes in relation to daily stressors and life events (122, 126–128). Further, given the expedited need for remote and digital assessment tools and telehealth provisions that have intensified due to the ongoing COVID-19 pandemic and its associated increase in mood symptoms and disorders (129–131), particularly BD, the additional value of longitudinal monitoring of rest-activity rhythms and its potential to flag possible preceding events for relapse and worsening of symptoms is immense (132–134).

As rest-activity changes can be robust in precipitating mood episodes, and are possible early indicators of impending episodes and of the beneficial effects of treatments, interventions targeted at regulating rest-activity patterns and those that integrate actigraphy over time may be especially effective in treatment and prevention strategies. Interpersonal and Social Rhythm Therapy (IPSRT), which has an SRT component designed to help individuals regularize their rest-activity pattern, is one of the few psychotherapies shown in clinical trials to be effective in treating BD (135–142). The rhythm regularization of IPSRT has been shown to reduce risk of recurrence over 2 years (143) with changes in regularity of daily routines mediating symptomatic outcomes, supporting rhythm regulation as a treatment target with potential for sustained benefits and prognosis improvements. Studies are underway with modified versions of SRT (144), for example a version delivered largely *via* telehealth that has shown promise in reducing mood symptoms and suicide propensity in BD (134).

### Future Directions

Combining digital monitoring, and holistic multi-modal assessment of dynamic symptomatology, with neuroimaging and cognitive assessments can help enable the field to uncover the neurobiological mechanisms responsible for the phenotypic presentations of BD. Larger scale studies will be especially helpful in elucidating heterogeneous aspects. As can be seen in Supplementary Table 2, several actigraphy devices are available and used for research purposes in clinical populations. Although these devices all estimate body movement through accelerometers on the *x*, *y*, and *z* planes, there can be heterogeneity in the types of actigraphy parameters extracted from the raw data. Table 1 outlines several different variables that can be referred to, e.g., when reporting disruptions in rest (e.g., SE, WASO, L_5_ activity). The field has yet to reach consensus on which parameters are key for our understanding of rest-activity patterns in BD. Going forward, optimal parameters and modalities should be further operationalized to ensure consistency in reporting across studies and to allow for the combined analysis of datasets. It should also be noted that estimating sleep *via* proxy markers, such as L_5_, can be problematic because of its derivative nature (73). Collaborative efforts such as those of the Motor Activity Research Network for Health (mMARCH) will also facilitate efforts to use common procedures and methods for data extraction and analysis. For example, a processing pipeline for actigraphy based on the GGIR package that was developed for mMARCH sites (145) extracts features of sleep, physical activity, and circadian rhythmicity, and applies JIVE to capture the joint variation across three domains. Consideration of the joint variation will lead to a better understanding of the interrelationships of rest, activity, and their rhythms within and between days. This package also includes standard methods for handling missing data and non-wear time that has not been systematically presented in most studies of actigraphy in BD. Variability of the features such as IV and IS, which capture regularity in activity patterns within and across days, respectively, as well as circadian features, will be particularly relevant to future studies of BD. These markers are key for their ability to address both rest and activity, as well as highlight the importance of variability across time – two aspects proposed to be fundamental to BD. Further, novel methods to assess this variability are still being developed and could be an important future methodological direction for study in this field (123). For full characterization of sleep, future studies should also use PSG and/or other well-established and more comprehensive sleep measures, as well as capture longer study periods and greater sample sizes.

## Conclusion

Our use of actigraphy as an avenue of investigation in BD continues to grow and holds enormous potential to transform our current understanding of the nature of BD and its treatment. Using digital technologies to capture temporally sensitive and nuanced change in rest-activity patterns will power our strides toward discovering new phenotypic markers for the illness and to targets for its detection, treatment, and prevention. The promise held by current findings is timely, as sophisticated developments in digital technologies and remote sensors are beginning to enable the collection and meaningful analysis of real-time longitudinal monitoring of complex mood disorders such as BD. Ideally, the guidelines outlined within the future directions section will afford researchers a framework for continued investigation of the temporally sensitive nature of rest-activity patterns, emphasizing the importance of pattern rhythmicity and regularity, with the goal of improving prognosis for those with and at-risk for BD.

## Data Availability Statement

The original contributions presented in the study are included in the article/Supplementary Material, further inquiries can be directed to the corresponding author/s.

## Author Contributions

PP, AS, and HB contributed to the conception and design of the review. PP and GdQC conducted the systematic search. PP wrote the first draft of the manuscript. GdQC, DG, and HB wrote sections of the manuscript. All authors contributed to manuscript revision, read, and approved the submitted version.

## Author Disclaimer

The views and opinions expressed in this article are those of the authors and should not be construed to represent the views of any of the sponsoring organizations, agencies, or the United States government.

## Conflict of Interest

HS receives royalties from Wolters Kluwer, royalties and an editorial stipend from American Psychiatric Association Press, and honoraria from Novus Medical Education and Medscape. RA is an unpaid scientific advisor for Ksana Health. The remaining authors declare that the research was conducted in the absence of any commercial or financial relationships that could be construed as a potential conflict of interest.

## Publisher’s Note

All claims expressed in this article are solely those of the authors and do not necessarily represent those of their affiliated organizations, or those of the publisher, the editors and the reviewers. Any product that may be evaluated in this article, or claim that may be made by its manufacturer, is not guaranteed or endorsed by the publisher.
