# Supplementary material for: Toward a Digital Future in Bipolar Disorder Assessment: A Systematic Review of Disruptions in the Rest-Activity Cycle as Measured by Actigraphy
Source: Front Psychiatry. 2022 May 23;13:780726. doi: 10.3389/fpsyt.2022.780726 (PMC9167949; doi:10.3389/fpsyt.2022.780726)
Supplement: Supplementary file 1 [file Data_Sheet_1.docx]

Supplementary Materials

# Supplementary Table 1: Systematic search strategy

| Databases searched | Date range searched | Primary search strategy | Seconday search strategy |
| --- | --- | --- | --- |
| PubMed/MEDLINE | From inception to 19^th^ February 2021 | bipolar disorder OR bipolar  actigraphy OR actigraph OR actimetry OR accelerometry OR smart watch OR health watch | acceleromet*  activity monitor*  monitoring, ambulatory  physical activity device |
| PsycINFO | From inception to 19^th^ February 2021 |  | acceleromet*  activity monitor*  wearable devices  physical activity device |
| EMBASE | From inception to 19^th^ February 2021 |  | acceleromet*  activity monitor*  physical activity device |

# Supplementary Table 2: Characteristics of studies reviewed

| Study | Country | | N | Mood status | Comparison group(s) | Mean age ($\boldsymbol{\pm}\mathbf{SD)}$ | % Female | Rest-activity domain reported | Actigraphy variable used | Actigraphy device | Actigraphy duration |
| --- | --- | --- | --- | --- | --- | --- | --- | --- | --- | --- | --- |
|  | | **Question 1: What are the rest-activity patterns associated with adult BD?** | | | | | | | | | |
| Banihashemi et al. (2016) | Australia | | 236 | NR | HC, MDD, anxiety | Affective disorders: 35.7 $\pm$ 20.8, HC: 38.8 $\pm$ 21.4 | Affective disorders: 64.7, HC: 59.7 | Rest, daily activity | Activity count | Actiwatch-64/L/2/Spectrum | 5 - 22 days |
| Boudebesse et al. (2015) | France | | 55 | Euthymic | HC | BD: 53.5 $\pm$ 11.4 9, HC: 54.10 $\pm$ 9.11 | BD: 65.4, HC: 44.8 | Rest | SE, TST, SOL, WASO, IV, FI | AW-7, CamNtech | 21 days |
| Bradley et al. (2017) | UK | | 88 | Any | HC | BD: 46.8 $\pm$ 11.1, HC: 42.5 $\pm$ 11.9 | BD: 67.4, HC: 69 | Rest | Sleep onset, sleep offset, TST, TIB, SE, sleep duration | GENEActiv | 21 days |
| Cantisani et al. (2016) | Switzerland | | 61 | Depressed | HC, MDD | BD: 46.18 $\pm$ 11.21, MDD: 43.30 $\pm$ 14.03, HC: 41.05 $\pm$ 13.82 | BD: 68.1, MDD: 50.0, HC: 57.9 | Daily activity | Activity count | Actiwatch | 24 hours |
| Curtiss et al. (2019) | US | | 68 | Euthymic | HC | BD: 37.4 $\pm$ 10.5, HC: 33.3 $\pm$ 12.6 | BD: 62.5, HC: 52.8 | Daily activity | Activity count | Actiwatch | 8 weeks |
| Geoffroy et al. (2014) | France | | 55 | Euthymic | HC | BD: 53.50 $\pm$ 11.49, HC: 54.10 $\pm$ 9.11 | BD: 65, HC: 45 | Rest | TIB, sleep duration, sleep latency, WASO, SE, FI, activity count, IS, IV | W-7CamNtec | 21 days |
| Gershon et al. (2012) | US | | 68 | Euthymic | HC | BD: 34.7 $\pm$10.5, HC: 33.3 $\pm$12.6 | BD: 62.5, HC: 53.8 | Rest | SOL, WASO, terminal wakefulness, total wake time | Mini Mitter AW64 | 8 weeks |
| Gershon et al. (2018) | US | | 122 | Euthymic | HC | BD: 28.2 $\pm$ 11.5, HC: 27.9 $\pm$ 13.6 | BD: 67.2, HC: 50.8 | Rest | Sleep onset, sleep offset, midpoint | AW64 Philips Actiwatches | 25-50 days |
| (Harvey et al. (2005) | UK | | 60 | Euthymic | HC, Insomnia | BD: 39.6 $\pm$ 15.2, Insomnia: 39.6 $\pm$10.6, HC: 35.0 $\pm$13.4 | BD: 50, Insomnia: 55, HC: 65 | Rest, daily activity | SOL, WASO, TST, daytime activity count | Ambulatory Monitoring  Actigraph | 8 days |
| Hauge et al. (2011) | Norway | | 81 | Depressed | HC, MDD, schizophrenia | Mood disorders: 42.9 $\pm$ 10.7, schizophrenia: 47.4 $\pm$ 11.1, HC: 38.2 $\pm$ 13.0 | Mood disorders: 44, schizophrenia: 12.5, HC: 62.5 | Daily activity | Activity count | Actiwatch | 2 weeks |
| Hori et al. (2016) | Japan | | 40 | Depressed | HC, MDD | Mood disorder: 38.5 $\pm$ 12.4, HC: 41.1 $\pm$ 15.0 | Mood disorder: 50, HC: 55 | Rest, daily activity | TST, WASO, FI, SOL, activity count | Micromini-Motionlogger Actiwatch | 7 days |
| Ihler et al. (2020) | France | | 196 | Euthymic | HC | BD: 45.95 $\pm$13.03, HC: 36.24 $\pm$13.54 | BD: 61.7, HC: 58.7 | Rest | TST, sleep latency, SE | Actiwatch AW-7 | 21 days |
| Jacobson et al. (2019) | Norway | | 55 | NR | HC, MDD | BD & MDD: 42.8 $\pm$ 11.0. HC: 38.2 $\pm$ 13.0 | BD & MDD: 43, HC: 62.5 | Rest, daily activity | Activity count | Actiwatch | Up to 2 weeks |
| Janney et al. (2014) | US | | 180 | Euthymic, or mild symptoms | Users of Mental Health Services (MHS), Non-users of MHS | 45.3 $\pm$12.2 | 65 | Daily activity | Activity count | ActiGraph AM-7164 | 7 days |
| Jones et al. (2005) | UK | | 38 | Euthymic | HC | BD: 44.37 $\pm$13.10, HC: 46.89$\pm$14.82 | BD: 73.7,  HC: 73.7 | Rest, daily activity | IV, IS, RA, activity count, SOL, sleep duration, SE, sleep fragmentation, time awake | Actiwatch | 7 days |
| Kaufmann et al. (2018) | US | | 122 | Euthymic | HC | BD: 28.2 $\pm$ 11.5, HC: 27.9 $\pm$ 13.6 | BD: 67.2, HC: 50.8 | Daily activity | Daytime midpoint | AW64 & Spectrum Pro | 25-50 days |
| Knapen et al. (2020) | The  Netherlands | | 255 | Euthymic | HC, unaffected siblings | BD: 50.3 $\pm$ 11.3, unaffected siblings: 54.3 $\pm$ 12, HC: 47 $\pm$ 16.3 | BD: 59, unaffected siblings: 60, HC: 54 | Daily activity | Activity count | Actiwatch 2 & Motionwatch 8 | 2 weeks |
| Krane-Gartiser et al. (2014) | Norway | | 58 | Manic, depressed | HC | BD mania: 51.2 $\pm$ 15.4, BD depression: 39.9 $\pm$ 15.6, HC: 41.7 $\pm$ 11.6 | BD mania: 61,  BD depression: 58, HC: 46 | Daily activity | Activity count | Actiwatch | 24 hours |
| Krane-Gartiser et al. (2017) | Norway | | 88 | Depressed, manic, mixed | MDD | BD depressed: 35 (27, 56), BD manic: 50 (42, 67), BD mixed: 41 (28, 57), MDD: 40 (30, 52)  (median (IQR)) | BD depressed: 58, BD manic: 61, BD mixed: 50,  MDD: 50 | Daily activity | Activity count | Actiwatch Spectrum | 24 hours |
| Krane-Gartiser et al. (2018) | Norway | | 99 | Manic | Schizophrenia, MDD, HC | BD mania: ﻿51.2 $\pm$15.4 11, MDD: ﻿43.8 $\pm$ 15.9, schizophrenia: ﻿41.5 $\pm$11.5, HC: ﻿41.7 $\pm$11.6 | BD mania: ﻿61, MDD: ﻿60, schizophrenia: ﻿54, HC: ﻿46 | Daily activity | Activity count | ActiwatchSpectrum | 24 hours |
| Krane-Gartiser et al. (2019) | France | | 122 | Euthymic | HC | BD: 38 (31, 54), HC: 34 (28, 53) (median (IQR)) | BD: 54, HC: 54 | Rest, daily activity | TST, SOL, WASO, SE, FI, IS, IV, M10 | AW-7 CamNtech | 21 days |
| Krishnamurthy et al. (2018) | US | | 54 | Manic, mixed, depressive | HC | BD: 35.5 $\pm$ 11.2, HC: 32.6 $\pm$ 11.2 | BD: 50, HC: 57 | Rest | TST, sleep latency, SE, number of awakenings, duration of awakenings | NR | 7 days |
| Lee et al. (2021) | US | | 123 | Depressed, manic | HC | BD: 48.1 $\pm$ 9, HC: 48.3 $\pm$ 8.3 | BD: 62, HC: 58 | Rest | TST, PS, bedtime, wake time | Actisleep-BT | 7 nights |
| McGlinchey et al. (2014) | US | | 68 | Euthymic | HC | BD: 34.7 $\pm$ 10.5, HC: 33.3 $\pm$ 12.6 | BD: 62.5, HC: 52.8 | Rest, daily activity | Activity count, total wake time, SOL, WASO, terminal wakefulness | Mini Mitter AW64 | 1 month |
| McGowan et al. (2019) | UK | | 87 | Euthymic | HC, BPD | BD: 39.23 $\pm$12.24, BPD: 34.14 $\pm$10.5, HC: 39.46 $\pm$12.51 | BD: 67.7,  BPD: 90.5,  HC: 68.6 | Rest, daily activity | M10, L5, RA, IS, IV | GENEActiv | 28 days |
| McGowan et al. (2020) | UK | | 87 | Euthymic | HC, BPD | BD: 39.2 $\pm$ 12.2, BPD: 34.1 $\pm$ 10.5, HC: 39.5 $\pm$12.5 | BD: 67.7,  BPD: 90.5,  HC: 68.6 | Rest, daily activity | M10, L5, RA, IS, IV | GENEActiv | 28 days |
| Merikangas et al. (2019) | US | | 242 | NR | HC, MDD | 48 $\pm$ 16.9 | 61.9 | Rest, daily activity | Activity count, sleep duration | Philips Respironics Actiwatch | 14 days |
| Millar et al. (2004) | UK | | 38 | Euthymic | HC | BD: 47.3 $\pm$ 10.61, HC: 45.8 $\pm$ 10.93 | BD: 57.9  HC: 57.9 | Rest | Sleep duration, SE, sleep onset latency, WASO | Actiwatch-R AW2 | 5 days |
| Moon et al. (2016) | South Korea | | 44 | Manic | HC | BD: 30.42 $\pm$ 10.88, HC: 23.00 $\pm$ 3.57 | BD: 50, HC: 44 | Rest, daily activity | M10, L5, RA, TST, sleep onset latency, SE | Actiwatch-L | 1 week |
| Mukherjee et al. (2018) | US | | 49 | Manic, depressed, or mixed | HC | BD: 36.10 $\pm$ 11.33, HC: 31.57 $\pm$ 10.33 | BD: 48, HC: 57 | Rest | TST, bedtime variability, sleep latency, sleep duration, SE | Sleepwatch-O | 7 days |
| Ritter et al. (2012) | Germany | | 59 | Euthymic | HC, high risk | BD: 32.7 $\pm$ 10, high risk: 25.4 $\pm$ 3.6, HC: 28.3 $\pm$ 7.2 | BD: 40.9, high risk: 22.2, HC: 42.9 | Rest | TIB, sleep latency, activity, sleep duration, wake time, SE, ﻿Wake periods ≥3 min, ﻿Wake period/hour | SomnoWatch plus | 6 days |
| Ritter et al. (2016) | Germany | | 49 | Euthymic | HC | BD: 32.95 $\pm$ 10.19, HC: 28.65 $\pm$ 7.3 | BD: 61.9, HC: 57.1 | Rest | TIB, sleep duration | SomnoWatch plus | 5-7 days |
| Robillard et al. (2013) | Australia | | 32 | Depressed | MDD | BD: 22.8 $\pm$ 4.8, MDD: 21.8 $\pm$ 4.3 | BD: 78.6, MDD: 72.2 | Rest | Sleep onset, sleep offset | Actiwatch-64 | 7 days |
| Robillard et al. (2013) | Australia | | 95 | Depressed | HC, MDD | BD: 23.2 $\pm$ 4.3, MDD: 20.1 $\pm$ 4.7, HC: 24.8 $\pm$ 2.5 | BD/MDD: 62.7, HC: 60 | Rest | TIB, TST, WASO, SE | Actiwatch-64 | 7 days |
| Robillard et al. (2016) | Australia | | 50 | NR | MDD | BD: 22.5 $\pm$ 5.1, MDD: 20.9 $\pm$ 4.6 | BD: 76, MDD: 48 | Rest | TST, SE, acrophase, rhythmicity | Actiwatch-64/L/2 | 2 weeks |
| Salvatore et al. (2008) | US | | 67 | Manic, mixed, and euthymic | HC | BD: 44.4 $\pm$9.8, HC: 42.3 $\pm$10.8 | BD: 80.6, HC: 75.0 | Rest, daily activity | Mesor, amplitude, acrophase | AMA128K Mini-Motionlogger actigraphs | 72 hours |
| Schneider et al. (2020) | Czech Republic | | 50 | Euthymic | HC | BD: 39.72 $\pm$ 12.85, HC: 39.68 $\pm$ 11.19 | BD: 60, HC: 68 | Rest, daily activity | M10, L5, RA, ADA, ﻿AQA1–4, sleep duration, sleep onset, sleep offset, RSL, ISL, ﻿APSO, ﻿AASO | MINDPAX | 90 days |
| Shou et al. (2017) | US | | 339 | Euthymic | HC, MDD | BDI: 39.5 $\pm$ 14.1, BDII: 38.4 $\pm$ 17.1, MDD: 44.4 $\pm$ 18.6, HC: 42.2 $\pm$20.9 | BDI: 63.6, BDII: 64.5, MDD: 71.1, HC: 55.6 | Daily activity | Activity count | Actiwatch Spectrum | 2 weeks |
| Slyepchenko et al. (2019) | Canada | | 111 | Euthymic, depressed | HC, MDD | BD: 37 $\pm$17,  MDD: 39 $\pm$22.75, HC: 30 $\pm$20 | BD: 57.6,  MDD: 65.8,  HC: 50 | Rest, daily activity | TST, SOL, SE, WASO, mean mid-sleep time, IS, IV, L5, M10, RA | Actiwatch 2 | 15 days |
| St-Amand et al. (2013) | Canada | | 40 | Euthymic | HC, Insomnia | BD: 44.6 $\pm$ 11.0, Insomnia: 42.8 $\pm$15.9, HC: 47.15 $\pm$ 10.4 | BD: 50, Insomnia: 61.5, HC: 46.2 | Rest, daily activity | ﻿SOL, WASO, TST, SE | Mini Mitter | 14 days |
| Verkooijen et al. (2017) | The Netherlands | | 261 | Euthymic | HC, non-affected siblings | BD: 50.3 $\pm$ 11.6, siblings: 54.7 $\pm$12.1, HC: 6.8 $\pm$16.3 | BD: 56.1,  siblings: 60.8, HC: 51.2 | Rest | Sleep duration, sleep onset, sleep offset, SOL, SE, WASO, sleep inertia | Actiwatch 2 | 14 days |
|  | | **Question 2: What relationship do rest-activity patterns have with other characteristics of BD?** | | | | | | | | | |
| Benard et al. (2019) | France | | 236 | Euthymic | HC | BD SA: 47.24 $\pm$ 12.63, BD No SA: 44.77 $\pm$12.95, HC: 9.66 $\pm$13.37 | BD SA: 72, BD No SA: 54, HC: 54 | Rest, daily activity | TIB, sleep duration, wake time, SE, sleep latency, mean activity, FI, IV, L5 onset, M10 onset | AW-7CamNtech | 21 days |
| Bertrand et al. (2020) | Canada | | 76 | Euthymic, depressed, or hypomanic | N/A | 49 (19.75) (median (IQR)) | 49 | Rest | Sleep onset, sleep offset, sleep duration | GENEActiv | 2 weeks |
| Boland et al. (2015) | US | | 48 | Euthymic | HC | BD: 32.63 $\pm$ 11.61, HC: 30.96 $\pm$ 12.9 | BD: 62.5, HC: 58.3 | Rest | Sleep onset, sleep offset, SOL, WASO, sleep duration, SE | Actiwatch AW-64 | 7 days |
| Bracht et al. (2018) | Switzerland | | 38 | Depressed | HC | BD: 47.6 $\pm$ 10, HC: 47.5 $\pm$ 11 | BD: 68.4, HC: 68.4 | Daily activity | Activity count | Actiwatch | 24 hours |
| Bradley et al. (2020) | UK | | 88 | Any | HC | BD: 46.8 $\pm$ 11.1, HC: 42.8 $\pm$ 11.9 | BD: 67.4, HC: 69.4 | Rest | Sleep onset, sleep offset, TST, TIB, SE, ﻿mean 24-hour sleep duration | GENEActiv | 21 days |
| Brochard et al. (2018) | France | | 67 | Euthymic | N/A | 43.8 $\pm$ 13 | 58.2 | Rest, daily activity | SE, sleep latency, FI, M10 onset, amplitude | Actiwatch AW-7 | 21 days |
| Eidelman et al. (2012) | US | | 73 | Euthymic | HC | BD: 34.66 $\pm$ 10.03, HC: 32.85 $\pm$ 12.91 | BD: 57.1, HC: 51.3 | Rest | Sleep onset, sleep offset, TST | MiniMitter AW64 | 28 days |
| Gershon et al. (2016) | US | | 76 | Euthymic | HC | BD: 34.4 $\pm$ 10.4, HC: 32.8 $\pm$ 12.5 | BD: 62, HC: 51 | Daily activity | Activity count | AW63 Actiwatches | 6 weeks |
| Gonzalez et al. (2014) | US | | 42 | Depressed, manic | N/A | 41.0 $\pm$ 11.2 | 64 | Daily activity | ﻿Activity count | Basic Motionlogger | 7 days |
| Gonzalez et al. (2018) | US | | 105 | Depressed, manic | N/A | 41.20 $\pm$ 11.34 | 62.9 | Rest, daily activity | Activity count, sleep percent, SE, sleep duration, IS, IV, RA | Basic Motionlogger | 7 days |
| Gross et al. (2020) | France | | 147 | Euythmic | N/A | 45 $\pm$ 13 | 60 | Rest, daily activity | Sleep duration, sleep latency, WASO, SE, FI, M10 onset, IS, IV, RA | Actiwatch AW-7 | 21 days |
| Kaplan et al. (2015) | US | | 159 | Euthymic | N/A | 35.8 $\pm$ 11.4 | 65.6 | Rest | TST, TIB | Actiwatch AW-64 | 7 days |
| Klein et al. (1992) | US | | 10 | Euthymic | N/A | 49.1 $\pm$ 12.4 | 40 | Rest, daily activity | Activity count, sleep duration, SE | NR | 6 days |
| Krane-Gartiser et al. (2016) | Norway | | 43 | Euthymic | N/A | Stable rest-activity group: 48.5 (43.8-58.8), unstable rest-activity group: 33 (25-46) (median (IQR)) | Stable rest-activity group: 86.3, unstable rest-activity group: 61.9 | Rest, daily activity | Activity count, TST, SE | Actiwatch Spectrum | 7 days |
| McKenna et al. (2014) | US | | 28 | Euthymic | HC | BD: 49.07 $\pm$ 11.34, HC: 46.36 $\pm$ 15.04 | BD: 78.6, HC: 71.4 | Rest, daily activity | ﻿Activity count, TST, SE | Respironics | 7 days |
| Ortiz et al. (2016) | Canada | | 20 | Euthymic | N/A | 51.6 $\pm$ 12.7 | 55 | Daily activity | Activity count | MicroMini Motionlogger | 2 weeks |
| Prunas et al. (2019) | France | | 115 | Euthymic | N/A | 46.25 $\pm$ 12.99 | 70 | Rest, daily activity | Sleep duration, sleep latency, WASO, SE, FI, IS, IV, RA, M10 onset | AW-7 CamNtech | 21 days |
| Scott et al. (2017) | Norway | | 34 | Manic, depressed, or mixed | N/A | 44.6 | 56 | Daily activity | Activity count | Actiwatch | 24 hours |
| Verkooijen et al. (2017) | The Netherlands & US | | 106 | Euthymic | HC | BD: 49.5 $\pm$ 11.4, HC: 45.5 $\pm$ 15.8 | BD: 45.1, HC: 54.5 | Rest, daily activity | Sleep duration, sleep onset, sleep offset, SOL, SE, WASO, sleep inertia, mean activity level, IS | Actiwatch 2 | 14 days |
|  | | **Question 3: What effect do interventions have on rest-activity patterns in BD?** | | | | | | | | | |
| Baune et al. (2006) | Australia | | 10 | Depressed | MDD | 48 $\pm$15 | 80 | Rest, daily activity | L5, M10, RA, IS, IV | Actiwatch-S | 14 days |
| Benedetti et al. (2007) | Italy | | 39 | Depressed | Sleep deprivation and light therapy responders or non-responders | Responders: 44.92 $\pm$13.08, non-responders: 46.69 $\pm$13.46 | Responders: 61.5, non-responders: 38.5 | Rest, daily activity | Amplitude, sleep onset, TST, time asleep at night, time awake at night, sleep ratio, sleep offset | Mini Motionlogger | 7 days |
| Esaki et al. (2019) | Japan | | 175 | Depressed, manic | Light and dark groups | 45.5 $\pm$ 13.1 | 53.7 | Rest | TST, SE, WASO, SOL, sleep midpoint | Actiwatch Spectrum Plus | 7 days |
| Etain et al. (2021) | France | | 90 | Euthymic | BDI or BDII | BDI: 42.71 $\pm$12.95, BDII: 48.35 $\pm$12.41 | BDI: 57, BDII: 70 | Rest, daily activity | TST, SOL, WASO, SE, FI | AW-7 | 21 days |
| Henriksen et al. (2020) | Norway | | 20 | Manic | BB glasses or placebo | BB glasses: 43.9 $\pm$11.8, placebo: 48.8 $\pm$14.1 | BB glasses: 40, placebo: 20 | Rest | SE, motor activity during sleep, TST, WASO, number of wake episodes, FI, sleep onset, sleep offset, mid-time sleep | Actiwatch Spectrum | 7 days |
| Hwang et al. (2017) | South Korea | | 25 | Depressed | Quetiapine XR or lithium monotherapy groups | Quetiapine XR: 37.2 $\pm$ 11.6, lithium: 32.7 $\pm$ 8.7 | Quetiapine XR: 70, lithium: 40 | Daily activity | ﻿Activity count | Actiwatch 2 | 8 weeks |
| Kaplan et al. (2018) | US | | 40 | Euthymic | BD-specific modification of CBT-I (CBTI-BP) with RISE-UP or psychoeducation (PE) comparison condition | RISE-UP: 39.3 $\pm$ 14.2, PE: 35.4 $\pm$ 8.9 | RISE-UP: 70, PE: 65 | Rest, daily activity | Activity count | Actiwatch AW-64 | 14 days |
| Kim et al. (2014) | South Korea | | 29 | Depressed | Lithium or quetiapine XR groups | Lithium: 33.9 $\pm$8.9, quetiapine XR: 39.2 $\pm$11.9 | Lithium: 41.2, quetiapine XR: 75.0 | Rest | SE, sleep latency, WASO, daytime activity count | Actiwatch 2 | 8 weeks |
| Scott et al. (2020) | France | | 70 | Euthymic | Lithium good-responders or non-responders | 41.3 (33, 52) (median (IQR)) | 57 | Rest, daily activity | TST, SOL, WASO, SE, FI, IS, IV, L5, M10, RA, IS, IV, amplitude | AW-7 | 21 days |
| Todder et al. (2006) | Australia | | 54 | Depressed | HC, MDD | Mood disorder patients: 49.5 $\pm$12.8, HC: NR | Mood disorder patients: 48.1, HC: 44.4 | Rest | TST, percentage sleep, SE, sleep latency | Actiwatch-S | Mood disorder patients: 14 days, HC: 7 days |

NR = not reported. SD = standard deviation. IQR = interquartile range. BD = bipolar disorder. HC = healthy control. MDD = major depressive disorder. BPD = borderline personality disorder. MHS = Mental Health Services. SA = suicide attempt. CBT-I = Cognitive Behavioral Therapy for Insomnia. RISE-UP = routine using CBT-I to address sleep inertia. PE = psychoeducation. SOL = sleep onset latency. FI = fragmentation index. TST = total sleep time. TIB = time in bed. WASO = wake after sleep onset. IS = interdaily stability. IV = intradaily variability. ADA = ﻿average daily activity. ﻿AQA1–4 = ﻿average activity in quarters of a day. ﻿RSL = ﻿the percentage of minutes within the main sleep, above preset inactivity threshold. ﻿ISL = ﻿the percentage of minutes within the main sleep, below preset activity threshold. ﻿APSO = ﻿average activity 2 hours prior to sleep onset. ﻿AASO = ﻿average activity ﻿2 hours after sleep onset. PS = ﻿percentage of time in bed spent sleeping.

# Supplementary Figure 1: Risk of bias assessment for intervention studies


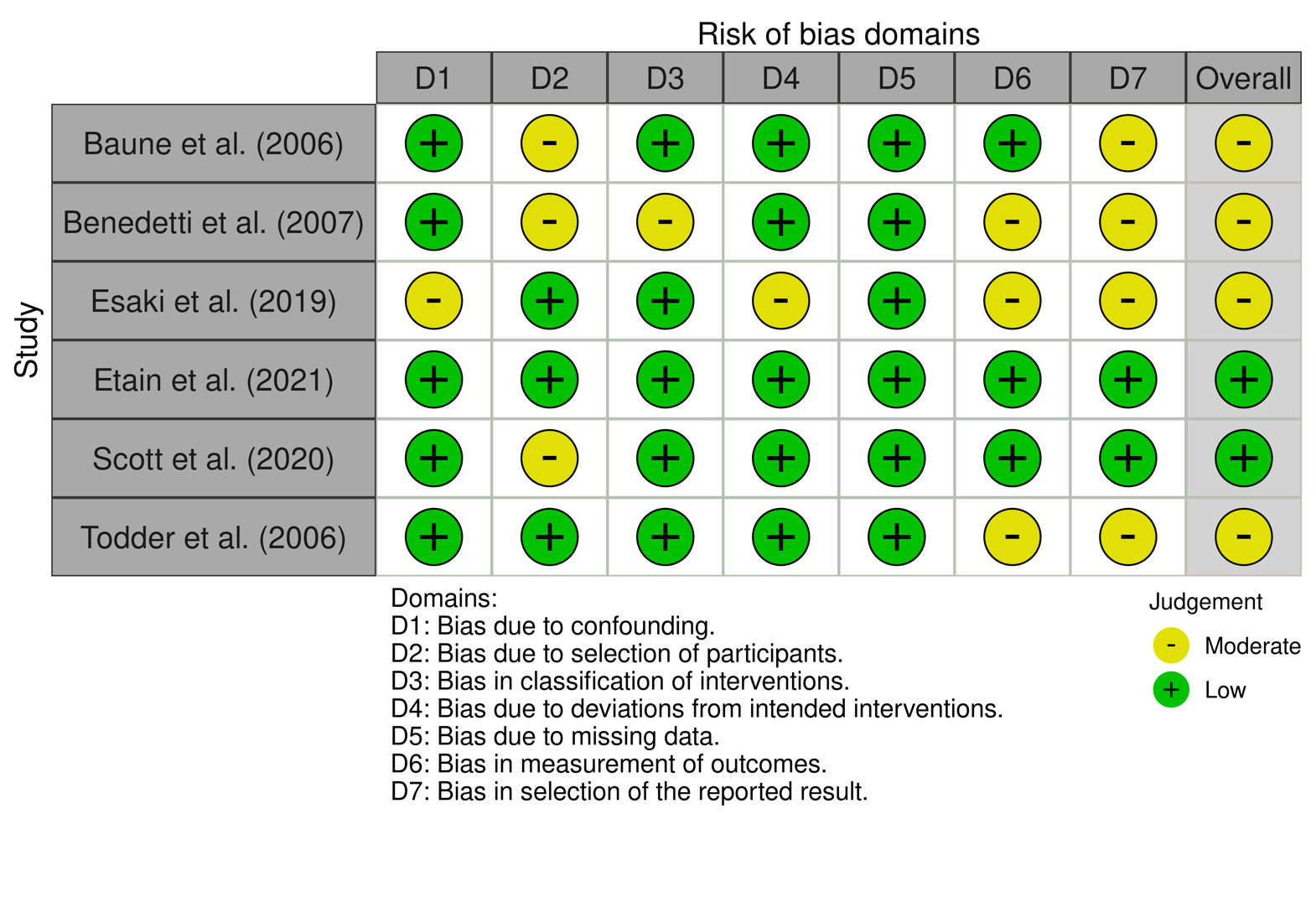


**A**

Supplementary Figure 1A: Risk of bias assessment using the ROBINS-I (Risk Of Bias In Non-randomised Studies - of Interventions) tool (Sterne et al., 2016). Produced using the Risk-of-bias VISualization (robvis) package (McGuinness & Higgins, 2021).


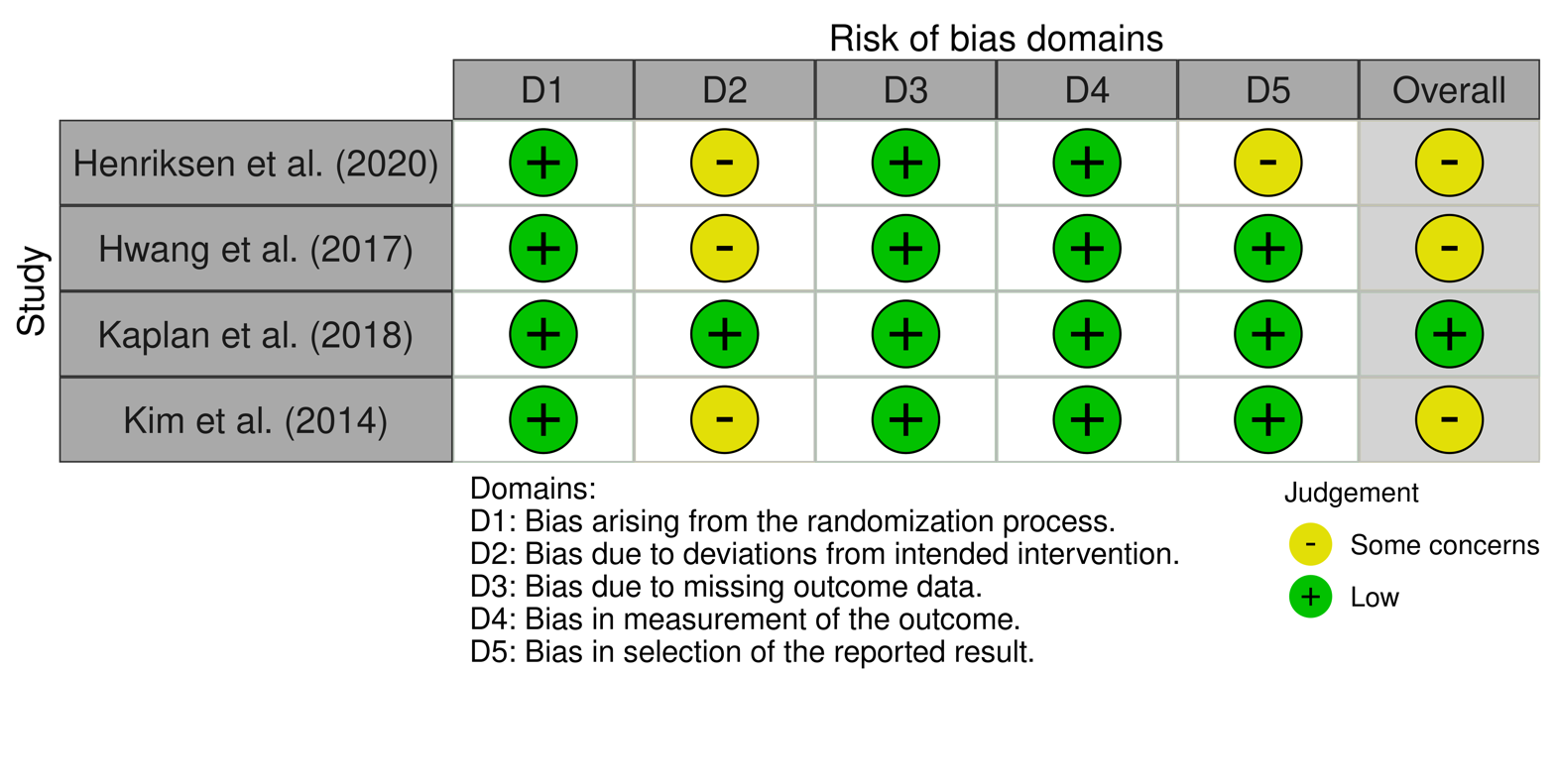


**B**

Supplementary Figure 1B: Risk of bias assessment using the RoB-2 (Risk of Bias in randomised trials) tool (Sterne et al., 2019). Produced using the Risk-of-bias VISualization (robvis) package (McGuinness & Higgins, 2021).

# References

Baune, B. T., Caliskan, S., & Todder, D. (2006). A case series on the development of rest-activity rhythm and quality of sleep in patients hospitalized for treatment of uni- or bipolar depression: A potential role for quetiapine. *International Journal of Psychiatry in Clinical Practice*, *10*(4), 269–275. https://doi.org/10.1080/13651500600736726

Benedetti, F., Dallaspezia, S., Fulgosi, M. C., Barbini, B., Colombo, C., & Smeraldi, E. (2007). Phase Advance Is an Actimetric Correlate of Antidepressant Response to Sleep Deprivation and Light Therapy in Bipolar Depression. *Chronobiology International*, *24*(5), 921–937. https://doi.org/10.1080/07420520701649455

Esaki, Y., Kitajima, T., Obayashi, K., Saeki, K., Fujita, K., & Iwata, N. (2019). Light exposure at night and sleep quality in bipolar disorder: The APPLE cohort study. *Journal of Affective Disorders*, *257*, 314–320. https://doi.org/10.1016/j.jad.2019.07.031

Etain, B., Meyrel, M., Hennion, V., Bellivier, F., & Scott, J. (2021). Can actigraphy be used to define lithium response dimensions in bipolar disorders? *Journal of Affective Disorders*, *283*, 402–409. https://doi.org/10.1016/j.jad.2021.01.060

Henriksen, T. E. G., Grønli, J., Assmus, J., Fasmer, O. B., Schoeyen, H., Leskauskaite, I., … Lund, A. (2020). Blue-blocking glasses as additive treatment for mania: Effects on actigraphy-derived sleep parameters. *Journal of Sleep Research*, *29*(5), e12984. https://doi.org/10.1111/jsr.12984

Hwang, J. Y., Choi, J.-W. W., Kang, S.-G. G., Hwang, S. H., Kim, S. J., & Lee, Y. J. (2017). Comparison of the effects of quetiapine XR and lithium monotherapy on actigraphy-measured circadian parameters in patients with bipolar II depression. *Journal of Clinical Psychopharmacology*, *37*(3), 351–354. https://doi.org/10.1097/JCP.0000000000000699

Kaplan, Katherine A., Talavera, D. C., & Harvey, A. G. (2018). Rise and shine: A treatment experiment testing a morning routine to decrease subjective sleep inertia in insomnia and bipolar disorder. *Behaviour Research and Therapy*, *111*, 106–112. https://doi.org/10.1016/j.brat.2018.10.009

Kim, S. J., Lee, Y. J., Lee, Y. J. G., & Cho, S. J. (2014). Effect of quetiapine XR on depressive symptoms and sleep quality compared with lithium in patients with bipolar depression. *Journal of Affective Disorders*, *157*, 33–40. https://doi.org/10.1016/j.jad.2013.12.032

McGuinness, L. A., & Higgins, J. P. T. (2021). Risk-of-bias VISualization (robvis): An R package and Shiny web app for visualizing risk-of-bias assessments. *Research Synthesis Methods*, *12*(1), 55–61. https://doi.org/10.1002/jrsm.1411

Scott, J., Hennion, V., Meyrel, M., Bellivier, F., & Etain, B. (2020). An ecological study of objective rest-activity markers of lithium response in bipolar-I-disorder. *Psychological Medicine*, 1–9. https://doi.org/10.1017/S0033291720004171

Sterne, J. A. C., Savović, J., Page, M. J., Elbers, R. G., Blencowe, N. S., Boutron, I., … Higgins, J. P. T. (2019). RoB 2: A revised tool for assessing risk of bias in randomised trials. *The BMJ*, *366*. https://doi.org/10.1136/bmj.l4898

Sterne, J. A., Hernán, M. A., Reeves, B. C., Savović, J., Berkman, N. D., Viswanathan, M., … Higgins, J. P. (2016). ROBINS-I: A tool for assessing risk of bias in non-randomised studies of interventions. *BMJ (Online)*, *355*. https://doi.org/10.1136/bmj.i4919

Todder, D., Caliskan, S., & Baune, B. T. (2006). Night locomotor activity and quality of sleep in quetiapine-treated patients with depression. *Journal of Clinical Psychopharmacology*, *26*(6), 638–642. https://doi.org/10.1097/01.jcp.0000239798.59943.77
